# Vitamin-Responsive Epileptic Encephalopathies in Children

**DOI:** 10.1155/2013/510529

**Published:** 2013-07-25

**Authors:** Satish Agadi, Michael M. Quach, Zulfi Haneef

**Affiliations:** ^1^Department of Pediatrics, Baylor College of Medicine, Houston, TX 77030, USA; ^2^Department of Neurology, Baylor College of Medicine, Houston, TX 77030, USA; ^3^Texas Children's Hospital, 6621 Fannin Street, CCC 1250.03, 12th Floor, Houston, TX 77030, USA

## Abstract

Untreated epileptic encephalopathies in children may potentially have disastrous outcomes. Treatment with antiepileptic drugs (AEDs) often may not control the seizures, and even if they do, this measure is only symptomatic and not specific. It is especially valuable to identify potential underlying conditions that have specific treatments. Only a few conditions have definitive treatments that can potentially modify the natural course of disease. In this paper, we discuss the few such conditions that are responsive to vitamin or vitamin derivatives.

## 1. Introduction 

Epileptic encephalopathies (EEs) are conditions in which progressive cognitive and neuropsychological regression occurs, attributable to excessive ictal and interictal epileptogenic activity during brain maturation [1]. The progression in such disorders is mostly relentless and leads to irreversible damage to the developing brain. Original classification of International League Against Epilepsy (ILAE) included only a few conditions under strict criteria of EE; however, in 2010, they extended the definition to any form of epilepsy that can cause encephalopathic effect [2]. Most of these conditions are managed symptomatically with AEDs; very rarely do these conditions have treatable underlying causes, including genetic, metabolic, autoimmune, and nutritional causes. Treatment with a specific vitamin or vitamin derivative in these specific cases may halt such inexorable progression.

## 2. Pyridoxine Dependent Epilepsy (PDE)

Hunt and colleagues reported the first case of intractable epilepsy in an infant controlled by pyridoxine in 1954 [3]. Subsequently, many anecdotal case reports surfaced [4, 5]. For a while, it was speculated that a mutation affecting Glutamate decarboxylase (GAD) was the cause for PDE. However, Battaglioli and colleagues showed that GAD mutation is not linked to PDE [6]. In 2006, Mills et al. for the first time reported that alpha aminoadipic semialdehyde dehydrogenase (antiquitin) deficiency due to ALDH1A7 mutation is a cause for PDE [7]. It usually manifest in neonatal period. The affected neonates usually manifest within the first few hours after birth with seizures. The seizure evolves into status epilepticus despite adequate treatment with AEDs. An antenatal history of unusual fetal movements indicating intrauterine seizures may be present although this is not very common. The seizure semiology is quite variable with focal, generalized, myoclonic, epileptic spasms, and/or mixed seizure patterns. PDE can be easily confused with hypoxic ischemic encephalopathy or sepsis due to age of onset and frequent seizure manifestation [8–10]. Rarely, the clinical manifestations may be delayed into the later part of infancy up to 2 months of age or beyond. Again, these children manifest with medically refractory epilepsy that can evolve into status epilepticus [11–13]. Many patients have elevations of the indirect biomarker pipecolic acid (PA) in plasma and in CSF [14, 15]. Patients with PDE have elevations of alpha aminoadipic semialdehyde (AASA) in plasma, CSF, and urine that serves as a specific biochemical marker [15]. However, AASA assay is not yet commercially available. Usually, these biochemical findings persist even after years of effective treatment [15]. There are no specific radiological findings unique to PDE. However, infants with PDE often have variable degrees of brain atrophy, thinning of the corpus callosum, mega cisterna magna, progressive hydrocephalus, and focal cortical dysplasia. The neuroimaging findings are not correlated to biochemical or genetic abnormalities [9, 16–18]. If treated early in the course, the brain MRI may become normal [19]. MR spectroscopy may show decreased N acetyl-aspartate to creatine ratio in the cerebral cortex indicating neuronal loss [20]. EEG findings are nonspecific and abnormalities range from a mildly slow background activity to burst-suppression pattern. In between these two extremes, one may find generalized and multifocal epileptiform activity, bursts of high voltage slow waves, and hypsarrhythmia. The paroxysmal events and seizures frequently are not associated with EEG changes indicating that not all the events are epileptic [21, 22]. Mutations in the *ALDH7A1 *gene have been proven to be the molecular cause of PDE. This gene encodes the protein antiquitin, an aldehyde dehydrogenase (AASA) that functions within the cerebral lysine catabolism pathway. Homozygous or compound heterozygous *ALDH7A1 *mutations have been reported in patients with neonatal as well as late-onset PDE [7, 15, 23–27]. Treatment is with an initial dose of 50 to 100 mg of IV pyridoxine which can result in dramatic seizure control and EEG improvement [28]. Occasionally, some patients may require up to 500 mg in sequential dosing. These interventions should only be attempted in a controlled ICU setting, as intravenous pyridoxine is reported to cause rare instances of respiratory arrest. This should be followed by a maintenance dose of 15–18 mg/kg/day in two daily divided doses with a maximum daily dose of 500 mg [9, 29]. Although a simple test of simultaneous EEG and pyridoxine challenge to detect improvement could be very beneficial, it neither identifies nor excludes PDE. The patient with suspected PDE should receive pyridoxine until the diagnosis is fully excluded by metabolic and/or DNA analysis [30]. Peripheral neuropathy and dorsal root ganglionopathy have been reported as a side effect of high-dose pyridoxine therapy [31, 32]. In addition to pyridoxine, a lysine-restricted diet might be of beneficial effect in improving the long term developmental outcomes of children with PDE [33]. 

Although early diagnosis and treatment are very important, a wide variety of neurodevelopmental disabilities have been noted in patients with PDE irrespective of timing of initiation of treatment, indicating an underlying multifactorial etiology for developmental outcome. They range from expressive language deficits to cognitive dysfunction, obsessive compulsive disorder, and pervasive developmental disorder. There may be motor developmental delay with associated persistent mild reductions in tone [9, 23, 34, 35]. The overall outcome in PDE still remains poor, and individual outcome cannot be predicted by many evaluated characteristics, including timing of initiation of treatment [36]. Other very rare causes of pyridoxine-dependent epileptic encephalopathies are hypophosphatasia due to tissue nonspecific alkaline phosphatase (TNSALP) deficiency, mutation in phosphatidylinositol glycan anchor biosynthesis class V (PIGV) causing hyperphosphatasia, and hyperprolinemia type II secondary to pyrroline 5-carboxylate (P5CD) deficiency [37]. All these conditions are autosomal recessive, and the associated seizures respond to pyridoxine. 

## 3. Pyridoxal 5 Phosphate Dependent Epilepsy

This syndrome was first described in 2002 by Kuo and Wang [38]. Recently, mutations in the *PNPO *gene, which encodes pyridoxine 5′-phosphate oxidase, were demonstrated to be a cause for this autosomal recessive condition [39]. Pyridoxal 5 phosphate (PLP) dependent epilepsy is a different entity from PDE with distinct clinical features and neurophysiologic manifestations. Neonates born with PLP dependent epilepsy are invariably premature and have features that mimic organic acidemia immediately after birth. Hypoglycemia and lactic acidosis with intractable seizures are often seen. Antenatal history of fetal seizures is very common. The seizure semiology includes clonic or myoclonic jerks and complex ocular, facial, or orolingual movements. These infants are refractory to treatment with AEDs or pyridoxine. If untreated, these infants will die or have severe neurodevelopmental impairments. Affected patients respond gradually to pyridoxal 5 phosphate treatment [40–42]. Plasma aminoacidogram will reveal elevated glycine and threonine. CSF neurotransmitter studies show elevated L-DOPA and 3-methoxytyrosine; decreased homovanillic acid and 5-hydroxyindoleacetic acid [43, 44]. Although classically interictal, EEG demonstrates a burst suppression pattern indicative of Ohtahara syndrome. Rarely, the EEG may be normal [40]. Recently, Schmitt and colleagues reported that EEG during the seizures and/or paroxysmal events was surprisingly inconclusive. They observed continuous or intermittent focal or generalized discharges of sharp waves or rhythmic sharp theta waves during the seizures, but more frequently, there was no discernible ictal change. They concluded that lack of ictal EEG discharge during distinct paroxysmal events is perhaps more suggestive of PDE or PLP dependent epilepsy than the inconstantly obvious ictal discharges [22]. Genetically, PLP dependent epilepsy is related to *PNPO* gene mutation located on chromosome 17 and is inherited in an autosomal recessive manner. Most of the mutations change one amino acid in the pyridoxine 5′-phosphate oxidase enzyme, impairing its normal function. The resulting enzyme cannot effectively metabolize pyridoxine and pyridoxamine to produce PLP. A shortage of PLP can disrupt the function of many other proteins and enzymes [39]. Treatment with pyridoxine has no impact on clinical or EEG characteristics. However, parenteral PLP results in significant improvement. The typical dose of PLP is 30–50 mg/kg/day in 3-4 divided doses as an enteral preparation [40]. Early diagnosis and treatment are the most important predictor of outcome. Untreated cases have high mortality, and survivors are left with poor neurocognitive outcome [40, 42]. 

## 4. Folinic Acid Responsive Epilepsies

Folinic acid responsive epilepsies are also commonly known as cerebral folate deficiency syndromes. Although cerebral folate deficiency is associated with diverse neurological conditions, only a few conditions are responsive to treatment with folinic acid. These are usually mediated by either genetic or autoimmune mechanisms and cause low concentration of CSF 5-methyl tetrahydrofolate (MTHF). Wevers and colleagues initially reported a case of a severe isolated CNS folate deficiency in a patient with slowly progressive neurological disease and suggested a disturbed transfer of folate across the choroid plexus [45]. Subsequently, Ramaekers and colleagues published detailed clinical history, treatment, and outcome in affected patients [46, 47].

### 4.1. Autoimmune Folate Antibodies

 Clinical signs and symptoms typically begin at around 4 months of age with an onset of irritability and sleep disturbance. This is usually followed by the development of psychomotor retardation, seizures, dyskinesia, cerebellar ataxia, and spastic diplegia. Other signs include deceleration of head growth in the early stages of the disorder. In untreated cases, visual disturbances manifest after 3 years of age and sensorineural hearing loss after approximately 6 years of age. About one-third of patients may develop epileptic spasms, recurrent myoclonic-astatic seizures, absence seizures, and generalized tonic-clonic seizures. Thirty-five percent of the patients also have autistic spectrum disorders [46, 47]. CSF MTHF level is typically low. Autoantibodies to folate are often present in serum. Interestingly, erythrocyte folate is usually normal. EEG findings are nonspecific and vary from mild diffuse slowing, multifocal spike-wave discharges, and hypsarrhythmic background pattern to electrical status epilepticus in sleep [48].

Neuroimaging findings are nonspecific and MRI of the brain may reveal supra- and infratentorial atrophy.

### 4.2. FOLR1 Gene Mutation

This condition is due to mutation in the FOLR1 gene located on the long arm of chromosome 11. This leads to an impaired transport of folate to the central nervous system in turn causing disturbed myelin metabolism and neurodegeneration. In sharp contrast to the early presentation of autoimmune folate antibody EE, these children typically presented between late infancy to eight years of age [48, 49]. The affected children present with intractable myoclonic-astatic, myoclonic, and generalized tonic-clonic epilepsy. Other neurological manifestations include ataxia, developmental delay, hypotonia, and regression of developmental milestones. Children who present late in the second decade may exhibit features of severe polyneuropathy. These patients have almost undetectable levels of CSF 5 MTHF with a decrease in CSF MTHF concentration greater than 80% below the lower limit of the reference range [50]. The most common findings on EEG are diffuse slowing of the background activity and multifocal spikes [49]. Brain MRI most frequently shows delayed myelination or hypomyelination of the cerebral white matter with mild cerebral atrophy and very pronounced cerebellar atrophy [49]. The FOLR1 gene is located on chromosome 11q13.3-q14.1. Homozygous mutations or compound heterozygous mutations lead to an autosomal recessive disorder [49]. 

Both of these conditions respond to folinic acid 0.5–1 mg/kg/day. Folinic acid is available in L and D isomer forms. The L isomer form has been shown to be more clinically effective. It should be emphasized that treatment with folic acid is contraindicated as it may exacerbate CSF MTHF deficiency causing further clinical worsening [51]. Patients who are diagnosed and treated before the age of 6 years are likely to show a favorable and sometimes dramatic response with marked neurological recovery and cessation of seizures. The older group of children beyond the age of 6 years tends to show a more delayed response with incomplete neurological recovery. However, treatment with folinic acid is still helpful to prevent further deterioration in these patients [51]. 

## 5. Biotinidase Deficiency

Biotinidase deficiency is a biotin-responsive, autosomal recessive neurocutaneous metabolic disorder causing multiple carboxylase deficiency and presenting with seizures, hypotonia, visual/auditory symptoms, eczema, and alopecia. The enzyme biotinidase cleaves biocytin to biotin which serves as a cofactor for five biotin-dependent carboxylase enzymes: pyruvate carboxylase, propionyl-CoA carboxylase, beta-methylcrotonyl-CoA carboxylase, and two isoenzymes of acetyl-CoA carboxylase. These carboxylases play an important role in fatty acid synthesis, amino acid catabolism, and gluconeogenesis. When biotinidase activity is 10–30% of normal, it is said to be a partial deficiency and when it is less than 10%, it is termed profound deficiency [52]. The global incidence of biotinidase deficiency is 1 in 60,000 live births [52]. Consanguinity is associated with higher rates of up to 20% and are seen in regions such as Turkey and Saudi Arabia [53]. Untreated children usually have neurocutaneous features between 2 and 5 months of life [54] although presentation can be in late adolescence or adulthood [55]. The neurologic features in biotinidase deficiency are thought to result from aberrant myelination. The most common neurologic feature in untreated patients are seizures which occur in 70% of symptomatic children with severe biotinidase deficiency [56]. These could take the form of tonic-clonic, myoclonic, or partial seizures or present as infantile spasms [56]. Hypotonia is another frequent observation in infants with biotinidase deficiency. Presentation at a later age is with ataxia and developmental delay often with visual problems and hearing loss. When children remain asymptomatic till later childhood or adolescence, presentation can be with features such as rapid visual loss with scotomas, optic neuropathy, and spastic paraparesis, causing diagnostic confusion with juvenile multiple sclerosis [57]. Cutaneous findings include atopic/seborrheic dermatitis, alopecia including loss of eyebrows and eyelashes, hypopigmentation of skin or hair, and fungal skin infections [58–61]. Respiratory symptoms have also been described and include hyperventilation, laryngeal stridor, and apnea [62]. Hyperventilation could be due to metabolic acidosis (see the following) and can lead to coma and death. Laryngeal stridor is likely due to neurological involvement. Lactic acidosis is commonly, but not universally, seen in symptomatic children. Mild to moderate hyperammonemia is common, although this may not be present even in severely affected children. The most helpful metabolic abnormality for diagnosis is the presence of abnormal organic acid metabolites in serum and urine which are elevated due to mitochondrial carboxylase deficiency, most commonly 3-hydroxyisovalerate [57]. Other organic acids elevated include 3-methylcrotonylglycine and methyl citrate suggesting the possibility of multiple carboxylase deficiency [59]. CSF lactate and pyruvate may be elevated. Serum biotinidase level will be low and confirms the diagnosis. Biotinidase activity is measured either by a semiquantitative fluorometric method using Biotinyl-6-amidoquinoline as substrate, or by a semiquantitative colorimetric method using N-biotinyl-p-aminobenzoic acid as substrate [53]. Biotinidase deficiency can also be demonstrated in peripheral blood leukocytes, cultured skin fibroblasts, or amniotic fluid for prenatal diagnosis [63]. Neuroimaging findings include cerebral atrophy with ventriculomegaly, enlarged CSF spaces, and diffuse white matter changes which may be related to dysmyelination [59, 60]. Recurrent encephalopathy with vasogenic edema in the bilateral putamen and caudate nuclei, infra- and supratentorial cortex, and brainstem and atrophy in chronic disease has been reported [64]. MR spectroscopy can show decreased NAA peak, elevated lactate, and reversal of the choline/creatine ratio. These spectroscopic findings are similar to mitochondrial disorders such as Leigh disease or Alpers' disease [60]. EEG findings in biotinidase deficiency include mild background slowing, attenuated background, multifocal spikes consistent with early infantile encephalopathy, burst suppression pattern, asynchrony, and hypsarrhythmia [56, 57, 59, 60]. EEG could be normal in affected children. ERG and VEP studies are normal, while BAEP can show findings consistent with sensorineural hearing loss [60]. The biotinidase gene is localized to chromosome 3p25. At least 150 different mutations have been reported to be associated with biotinidase deficiency [62]. Treatment with biotin can prevent symptoms if biotinidase deficiency is detected in neonatal screening. If symptomatic, improvement is seen often within a day of treatment. The seizures often do not respond to AEDs, but quickly respond to biotin supplementation [56]. It has been suggested that, similar to pyridoxine trials, a trial of biotin should be considered in any child with poorly controlled seizures [57]. The resolution of symptoms with treatment can be rewarding, often in the face of multiple failed previous attempts without the correct diagnosis [59]. Regardless of age or weight, a dose of 5–20 mg daily has been found to be effective [59, 65] and needs to be continued lifelong. Noncompliance can result in recurrence of symptoms within weeks to months [53]. Holocarboxylase deficiency can present with symptoms similar to biotinidase deficiency and also responds to biotin supplementation [60].

With routine neonatal screening for the disorder [66], full blown cases of biotinidase deficiency are uncommon in the developed world. In parts of the world where universal screening is not available, physicians must screen all first-degree relatives for deficiency and treatment, as there is a high incidence in close relatives [53, 59]. Raw egg whites should be avoided as they contain avidin, which binds biotin and decreases its bioavailability [62]. Most of the clinical, EEG, and imaging changes can be reversible other than for sensorineural hearing loss [67], optic atrophy, and developmental delay [53, 59, 60, 62, 68]. 

## 6. Vitamin B12 Deficiency

Leichtenstern [69] and Lichtheim [70] gave the earliest accounts of the neurological associations of megaloblastic anemia as lesions [71, 72] in the posterior and lateral columns of the spinal cord. The term “subacute combined degeneration of the cord” (SCD) was coined by Russell et al. [73]. Folic acid was synthesized in 1945, and its rampant use in the treatment of megaloblastic anemia led to several cases of worsening of neurological complications. The clinical presentation of vitamin B12 deficiency may be with anemia or with neurological symptoms, the latter of which is discussed in the following. In patients with megaloblastic anemia, the frequency of neurological manifestations was found to be as follows: SCD (15%), dysautonomia, peripheral neuropathy (40%), and optic atrophy (2%). Psychiatric manifestations include mood and behavioral changes (20%), memory loss, and cognitive decline (25%) [74]. Peripheral neuropathy presents as distal symmetric sensory loss with ataxia. Large fiber symptoms such as loss of vibration and proprioception predominate. Reflexes may be increased, normal, or diminished depending on the degree of spinal cord involvement. Patients without anemia or macrocytosis tend to have the most severe nervous system manifestations [75]. 

In infants, maternal vitamin B12 deficiency is the most common cause and manifests in breast-fed infants between 4 and 8 months [76]. The mothers are often vegan as the vitamin is absent in plants. The onset is more rapid in infants (over months) than in adults (over years), and the manifestation is mostly in the central nervous system [77]. In infants, neurodevelopmental delay, regression of motor milestones, failure to thrive, irritability, apathy, hypotonia, hyperreflexia, tremor, choreoathetoid movements, and microcephaly can occur [71, 76–81]. Seizures with vitamin B12 deficiency are rare but have been reported especially in infants [78, 80, 82], including a case of West syndrome [83]. The potential mechanism of seizures has been suggested to be homocysteine toxicity as has been shown in rats, and infants may be predisposed due to an incompletely formed blood-brain barrier [84]. Seizures can also occur in adults [84, 85].

The neurotoxic mechanisms of vitamin B12 deficiency are not fully understood. Vitamin B12 is a key cofactor in two biochemical reactions: (1) synthesis of methionine from homocysteine by methionine synthase and (2) conversion of methyl malonyl CoA to succinyl CoA. Consequently, when vitamin B12 is deficient, there is accumulation of homocysteine and methylmalonic acid, both of which can be measured to diagnose vitamin B12 deficiency [76]. Methylcobalamin is required in the central nervous system for myelin synthesis, and deficiency is thought to cause dysmyelination manifesting as diffuse white matter/axon sheath involvement leading to encephalopathy, myelopathy, peripheral neuropathy, and optic neuropathy. If serum vitamin B12 levels are in the low normal range, methionine and methylmalonic acid measurements may be more useful for diagnosis. Additionally, reduced methylmalonyl CoA breakdown causes excess propionyl CoA leading to odd-chain fatty acid incorporation into nerve sheaths [86]. Elevated urine methylmalonic acid is the most important laboratory test to diagnose vitamin B12 deficiency at the tissue level [87, 88]. In infants with encephalopathy, brain atrophy is noted in initial scans and can improve after vitamin B12 supplementation [72, 78, 79, 89]. MRI of the spinal cord can show T2 hyperintensity in the posterior columns consistent with SCD [90].

EEG shows slowing with encephalopathy. Patients with seizures can have epileptic discharges in the EEG which may be diffuse [78] or focal [85]. A modified hypsarrhythmia pattern was seen in an infant presenting as West syndrome that did not respond to ACTH, but responded to B12 supplementation [83]. Peripheral nerve involvement leads to a pattern of axonal neuropathy in the EMG [74]. As with hematological treatment of vitamin B12 deficiency, neurological complications due to vitamin B12 deficiency are also treated with weekly injections of 1000 mcg of hydroxocobalamin or cyanocobalamin for 3 months, followed by maintenance injections every 3 months [75]. Lack of response in 3 months calls the diagnosis of B12 deficiency into question. Early treatment can prevent long-term sequelae and can even improve acute symptoms [72, 78, 85]. About 90% of patients have improvement in symptoms of 50% or more, and up to 10% can have residual moderate to severe disability [91]. Infants with vitamin B12 deficiency have a risk of poor intellectual outcome in long-term follow up [72, 77, 80].

## 7. Conclusion

Although most of the epileptic encephalopathies carry worse prognosis, only a few of them are responsive to vitamin and its derivatives and thereby alter the overall prognosis. This can certainly provide a ray of hope to these relentless progressive conditions. The treating physician should be able to timely identify and treat them to further minimize the morbidity and mortality. Here is a table of summary of all vitamin responsive epileptic encephalopathies with clinical, biochemical, genetic, neurophysiological findings and treatment with prognosis (Table 1).

## Figures and Tables

**Table 1:** Summary of clinical, biochemical, neurophysiological findings with treatment and prognosis of vitamin responsive epileptic encephalopathies in children.

	Usual age of onset	Etiology	Biochemical abnormalities	Type of epilepsy	EEG findings	Treatment	Prognosis
Pyridoxine dependent epilepsy	0–2 months	ALDH7A1 mutation	Elevated CSF/urine alpha aminoadipic semialdehyde; elevated CSF/plasma pipecolic acid	Focal or generalized, myoclonic, epileptic spasms	Normal; Mild background slowing; generalized and multifocal epileptiform activities; hypsarrythmia	Pyridoxine (IV followed by oral)	Variable, dependent on early treatment with pyridoxine

Pyridoxal 5-phosphate dependent epilepsy	Early neonatal	PNPO mutation	Hypoglycemia and lactic acidosis; elevated plasma glycine and threonine; elevated CSF L-Dopa and 3-methoxytyrosine; decreased CSF homovanillic acid and 5-hydroxyindoleacetic acid	Multifocal myoclonic-tonic	Multifocal sharp waves; Burst suppression	Pyridoxal 5′-phosphate (Oral)	High rate of mortality and poor neurocognitive outcome

Autoimmune folate antibody related epilepsy	~4 months	Folate antibody mediated	Decreased CSF 5-methyltetrahydrofolate; serum folate antibodies	Epileptic spasms, myoclonic-astatic seizures, absence, generalized tonic clonic	Mild diffuse slowing; multifocal spikes, hypsarrythmia, electrical status epilepticus of sleep	Folinic acid (oral)	Favorable outcome if treated before 6 years of age; incomplete neurological recovery if treated later

FOLR1 mutation related epilepsy	4–8 years	FOLR1 mutation	Decreased CSF 5-methyltetrahydrofolate	Myoclonic-astatic, myoclonic, generalized tonic clonic	Diffuse slowing with multifocal spikes	Folinic acid (oral)	Favorable outcome if treated before 6 years of age; incomplete neurological recovery if treated later

Biotinidase deficiency	2–5 months (Late onset adolescence to adulthood)	Biotinidase gene mutation	Decreased serum biotinidase; lactic acidosis; hyperammonemia; elevated 3-hydroxyisovalerate, 3-methylcrotonylglycine and methyl citrate; elevated CSF lactate and pyruvate	Generalized tonic clonic, myoclonic, partial seizures, infantile spasms	Normal; mild slowing; asynchrony, attenuated background; multifocal spikes; burst suppression; hypsarrythmia	Biotin (oral)	Improved with treatment

B12 deficiency	Infantile	Dietary	Elevated urine methylmalonic acid	Focal or generalized, epileptic spasms	Generalized slowing; focal or generalized epileptic discharges; hypsarrythmia	Hydroxycobalamin or cyanocobalamin (IM)	Preventable long-term sequelae with treatment
